# The Sussex County Hospital, Brighton

**Published:** 1890-04-19

**Authors:** E. F. Bourchier

**Affiliations:** Secretary, by Order of the Committee of Management


### The Sussex County Hospital, Brighton.

From Lieutenant-General E. F. Bourchier, Secretary, by Order of the Committee of Management.

The pamphlet reviewed in your paper of the 15th ult. was brought before the Committee of Management of the above institution, which I am to observe includes the whole of the honorary medical staff, but being written anonymously, they declined to take any notice of it. They, however, sent a message to the writer through a member of the committee, who was acquainted with him, that if he would tabulate the different charges he might have to prefer against the management of the hospital, and personally attend and favour the committee with the evidence he might wish to produce in support of them, the committee would be only too glad to inquire into them. He has failed to put in an appearance, and seemingly prefers to work in a more indirect way. As a copy of the pamphlet has fallen into your hands, and you have reviewed it, the committee would wish to offer the following remarks on your editorial:—

The allegations may be summarised thus—

1. That the average cost per bed in the Sussex County Hospital exceeds that of other similar institutions by £14 a-year.
2. That the out-patients' reception-room contrasts unfavourably with that of the Brompton Hospital, and especially in this, that the patients, whilst waiting, can obtain no refreshments.

   Further, that an offer to remedy this last defect having been made, it was, after long delay, declined.
3. That no entertainments, such as concerts, dramatic recitals, penny readings, and such like, are allowed.
4. That the corridors, offices, and bath-rooms are never warmed, and the wards and chapel inadequately so.
5. That little or no trouble is taken to collect the subscriptions of old subscribers, and new subscribers are unsought for.

1. As to the first of these it is not denied that the Sussex County Hospital is an expensive one; but it is contended that there is no extravagance. All that is spent is so for the greater comfort and more speedy restoration to health of the patients. At the same time the Committee of Management deprecate any hasty comparison with other Hospitals. The difference in the price paid for the chief articles of consumption, the cost of necessary repairs and alterations in any given year, the fact that the gross amount of expenditure shown in our accounts includes the cost of the out-patients' department, as also the charges of our small convalescent home—these points are apt to be overlooked and require that any average struck should be that of a series of years, say five or ten, rather than that of any single one. The committee would be glad for any one to show them how they could economise without injury to the efficiency of the hospital.

2. As to the out-patients' waiting-room, the committee believe it will not contrast unfavourably with those in similar institutions. With reference to a refreshment stall, the committee extract the following from their last annual report: "During the past year an anonymous offer was made to fit up a refreshment stall for the benefit of the out-patients, who necessarily often have to wait some time ere they can be seen, and to construct a lift free of expense to the hospital. The committee would gladly have availed themselves of both these generous gifts, and at once appointed sub-committees to determine the best sites for the refreshment stall and lift respectively. As to the first, no suitable site could be found. The accommodation for the out-patients inside the hospital is barely sufficient as it is, and affords no spare space for any such stall as proposed. Outside the hospital there was but one spot that could be deemed in the least eligible, and this was open to many objections, and not least the difficulty of access to it in all weathers. The committee, therefore, very regretfully found themselves unable to accept this offer. An effort was made to get one of the tea and coffee vendors on the cliff to come up for an hour or two in the day, but this was unsuccessful—it was thought it would not pay. As to the lift, the sub-committee met many times, and successively different sites were suggested, discussed, and rejected, but eventually they unanimously agreed to recommend one which it was hoped would meet with general approval. But meanwhile the intending benevolent donor withdrew his offer. It is feared that he did not fully realize the difficulties of introducing so large a piece of machinery as a lift into an old building, or the sacrifice of the much needed private and public accommodation this would entail. As the committee do not see their way to construct a lift at the cost of the hospital, the project is for the present in abeyance." It is to be regretted that out-patients are detained so long as they are, but this is unavoidable. Some come long before the medical officers arrive; then these have to see, it may be, 30 or 40 patients each, and finally all have to go to the dispensary for their medicine. Under such circumstances it is repeated, detention for some time is unavoidable. Many are thoughtful enough to bring a "sup and a bite" with them.

3. As to the want of entertainments, the committee consider they are as competent to form an opinion as to their expediency as the writer of the pamphlet, and they decidedly think that in a General Hospital they would be out of place. They make an exception at Christmas, when they have a Christmas tree for the children, and an entertainment for the convalescent patients.

4. In the same way as to the warming of the corridors. It may be very desirable in a Consumption Hospital such as that at Brompton to keep up an equal temperature throughout the building, but it would not be so in a General Hospital; neither has any complaint as to the coldness of the corridors or the w.c.'s been hitherto made to the committee. Besides, the patients as a body have no access to the corridors; only those convalescent pass along them, going out for a walk in the grounds. The wards and bath-rooms are warm enough, and as to the chapel, this is now heated by a large "Gill" stove, of power to warm a much larger room, but its heat is not equally diffused. The committee have under consideration how they can improve this.

5. As to subscribers, old and new, and the collection of subscriptions, some sixty per cent. pay their subscriptions in January, and others with commendable punctuality; it is the few only that require to be looked up, and this is done. Last year we had no fewer than 183 new subscribers.

The Committee have thus gone through the allegations, and confidently leave to others to say how far they are open to the animadversions passed on them by this anonymous writer; it being specially borne in mind that this Hospital having been erected some sixty years ago, all the committee are responsible for is the introduction of modern improvements to the extent of their pecuniary ability as these are from time to time suggested to them. They fearlessly challenge any investigation into their management of this valuable institution and would at any time gladly welcome a visit and inspection from any one interested in the matter, and more especially any person connected with the publication, The Hospital, in which this review appeared.

[We have much pleasure in publishing the above statement, and shall take an opportunity of inspecting the hospital, as the committee suggest.]

